# Patient Satisfaction and Long-Term Clinical Outcomes in Adolescent Sub-inguinal Microscopic Varicocelectomy

**DOI:** 10.7759/cureus.44349

**Published:** 2023-08-29

**Authors:** Joao G Porto, Adele Raymo, Maria Camila Suarez Arbelaez, Aaron A Gurayah, Ranjith Ramasamy

**Affiliations:** 1 Desai Sethi Urology Institute, University of Miami, Miami, USA; 2 Department of Pediatric Urology, Nicklaus Children's Hospital, Miami, USA

**Keywords:** hypogonadism, testicular hypotrophy, male infertility, varicocelectomy, adolescents, varicocele

## Abstract

Introduction and objective: The long-term outcomes of varicocelectomy in adolescents is debated. The aim of this study was to evaluate symptom improvement, hypogonadism, and paternity in those who underwent adolescent sub-inguinal microscopic varicocelectomy.

Material and methods: A retrospective chart review was done of adolescents (median=19, interquartile range (IQR)=16-19.75 years) who underwent microscopic varicocelectomy between 2011 and 2021. Demographics, surgical indications, and outcomes were collected, as well as pre- and postoperative hormone levels and semen parameters. A questionnaire was prospectively collected to evaluate orchialgia, paternity, and symptoms of hypogonadism. Descriptive statistics and t-tests were performed, with significance assessed at p-value < 0.05.

Results: A total of 46 adolescents were included. Age at the time of diagnosis and surgery was 19 (IQR=16-20) and 19 (IQR= 18-21) years, respectively. Follicle-stimulating hormone (FSH), luteinizing hormone (LH), and total testosterone were not affected after surgery. Similarly, semen parameters were preserved after varicocelectomy, with median concentration increasing from 12x10^6^/ml to 16x10^6^/ml but not reaching significance (p=0.272). A total of 26 men completed the questionnaire. The age of responders was 22 (IQR=21-24) years, and the time after having had the varicocelectomy was 36.5 (IQR= 18.25-62.25) months. Orchialgia persisted in five men, and three reported having a recurrence. Two men, who had a preoperative abnormal semen analysis, were actively trying to have children and reported successfully having achieved natural pregnancies. None of the patients reported having hypogonadism, and none were receiving testosterone therapy.

Conclusion: Our study suggests that microscopic varicocelectomy in adolescents appears to be a safe and feasible procedure with a low rate of syndrome recurrence and no association with symptoms or biochemical evidence of hypogonadism.

## Introduction

Varicoceles are enlargements of the veins located within the pampiniform plexus and are known to be associated with abnormal semen parameters, reduced testicular growth, orchialgia, and hypogonadism [1-3]. In adolescents, the prevalence of varicoceles is 7.8% in the age group of 11-14 years and 14.1% in the age group of 15-19 years [4]. The increase in frequency with age demonstrates the progressiveness of the disease; therefore, varicocele-related testicular hypotrophy is more commonly observed in older adolescents [4]. Although this evidence is well-documented and should prompt timely intervention, there is still significant disagreement regarding whether adolescents with varicocele benefit from surgical correction or should continue active surveillance instead.

Elder et al. explain that the lack of consensus on the appropriate intervention for adolescents with varicocele has perpetuated due to the conclusions of the initial studies on this topic [5]. For example, from 1989 to 2005, parents of adolescents with varicocele were allowed to choose between observation and treatment (antegrade sclerotherapy) [6]. Then in 2011, men who were over 30 years of age were contacted regarding paternity, time to conception, and whether they had visited a fertility center. The authors concluded that testicular volume discrepancy (TVD) and surgical intervention during adolescence were not associated with paternity rates, suggesting that observation, regardless of testicular hypotrophy, was feasible and safe in adolescents with varicocele. However, in a study of patients conducted between 1970 and 2015, it was found that men who had a prompt varicocelectomy had better fertility and fecundity outcomes than men who had surgery due to subfertility in adulthood [7].

Discrepancies in study results have also permeated the current guidelines about varicocele treatment in adolescents. The European Association of Urology (EAU)/European Society for Paediatric Urology Guidelines Panel found moderate evidence that varicocele treatment results in improvement of testicular volume and sperm concentration [8]. The evidence was merely moderate because of the highly variable surgical indications, definition of treatment success, and scarce studies assessing long-term clinical outcomes and paternity rates post varicocelectomy in this population [6,9,10]. The American Urology Association's best practice policy and American Society of Reproductive Medicine Practice Committee report stated that varicocelectomy should be performed in adolescents if abnormal semen analysis or/and reduced testicular size, ipsilateral to the varicocele, are observed [11]. Nonetheless, the lack of objective parameters for assessing testicular hypotrophy dilemma of whether semen analysis should be collected in adolescents, still leaves the surgery decision primarily to the judgment of the physician and parents [5].

This study aims to evaluate the short- and long-term outcomes of microscopic sub-inguinal varicocelectomy in adolescents. Secondarily, we wanted to analyze long-term patient satisfaction, symptom resolution, paternity, and prevalence of hypogonadism after undergoing varicocelectomy.

## Materials and methods

We conducted a retrospective review of data obtained from adolescents who underwent microscopic varicocelectomy between 2011 and 2021 at the University of Miami Hospital, University of Miami, Florida, United States. The study was approved by the Institutional Ethics Review Board for Clinical Research, University of Miami. The procedures were performed with active participation from residents and fellows. Inclusion criteria for participant selection were: (i) a primary diagnosis of varicocele, (ii) age between 12 and 21 years and Tanner stage V at the time of surgery, (iii) microscopic varicocelectomy performed more than one year ago, and (iv) at least one postoperative visit.

We retrospectively reviewed demographic data, operative variables, pre- and postoperative hormone levels of follicle-stimulating hormone (FSH), luteinizing hormone (LH), and testosterone, and semen parameters. Semen samples were collected after two to three days of sexual abstinence, and the basic sperm parameters were measured in the same andrology laboratory using phase-contrast microscopy, following the guidelines provided by the World Health Organization (WHO), with measurements taken in a controlled room temperature of 22°C [12]. The median time between surgery and the first postoperative semen analysis was four months (IQR=3-6).

In June 2022, we contacted all patients who met the inclusion criteria via phone and requested their participation in a questionnaire. The questionnaire consisted of six questions designed to evaluate different aspects related to the procedure, such as the presence of orchialgia and its laterality, varicocele recurrence, the need for surgical reintervention, paternity status, and the diagnosis and treatment of hypogonadism following varicocelectomy.

Initially, we conducted a descriptive analysis of the entire cohort to provide an overview of the study population. Subsequently, we performed an unpaired t-test to compare the differences in hormone levels and semen parameters before and after varicocelectomy. Additionally, we compared the characteristics of the overall cohort with those of the interviewed subgroup to assess the representativeness of the interviewed participants relative to the initial sample of 46 adolescents. For statistical analysis, we considered a threshold of p-value < 0.05 as indicative of statistical significance.

## Results

A total of 46 subjects met the inclusion criteria. The median age at the time of varicocele diagnosis was 19 years (IQR=16-19.75). White (93.48%) and Hispanic or Latino (67.39%) were the most common race and ethnicity, respectively. Overall, six (13.04%) adolescents reported having comorbidities such as psychogenic erectile dysfunction (ED), Crohn’s disease, or sickle cell trait, among others. None of the patients had previously conceived a child, and only two (4.35%) patients consulted due to infertility.

The first and second most common reasons for consultation were orchialgia (54.35%) and abnormal semen analysis (21.74%), respectively (Table 1). Most of the varicoceles were present only on the left side (71.74%) and were grade 3 (67.39%). TVD was noted by urologists in eight (17.39%) adolescents. Prior to the microscopic procedure, nine (19.56%) patients had already received treatment for varicocele. Among these individuals, six (66.67%) had undergone an open varicocelectomy, while three (33.34%) had undergone a laparoscopic varicocelectomy.

**Table 1:** Clinical characteristics of adolescents with varicocele TVD: Testicular Volume Discrepancy

Variables	Overall population (N=46)	Interviewed population (N=26)	p-value
Age at varicocele diagnosis (years)	19 (IQR: 16-19.75)	18 (IQR: 16-19)	0.710
Main complaint, n (%)			
Orchialgia	25 (54.35%)	11 (42.31%)	0.930
Incidental finding by physician	6 (13.04%)	5 (19.23%)	
Incidental finding by patient	4 (8.70%)	4 (15.38%)	
Infertility	2 (4.35%)	1 (3.85%)	
Abnormal semen analysis	10 (21.74%)	5 (19.23%)	
Varicocele recurrence	3 (6.52%)	2 (7.69%)	
Decrease testicular size	2 (4.35%)	1 (3.85%)	
Laterality, n (%)			
Left	33 (71.74%)	18 (69.23%)	0.822
Bilateral	13 (28.26%)	8 (30.77%)	
Grade of varicocele, n (%)			
G1	3 (6.52%)	2 (7.69%)	
G2	12 (26.09%)	6 (23.08%)	
G3	31 (67.39%)	18 (69.23%)	
Palpable TVD, n (%)			
Yes	8 (17.39%)	6 (23.08%)	0.558
No	38 (82.61%)	20 (76.92%)	
Prior surgery, n (%)			
No	37 (80.43%)	17 (65.38%)	0.840
Open	6 (13.04%)	6 (23.08%)	
Laparoscopic	3 (6.52%)	2 (7.69%)	

The median age of the men at the time of varicocelectomy was 19 (IQR=18-21) years. The main indication for surgery was orchialgia (39.13%), followed by abnormal semen analysis (28.6%). All varicocelectomies were performed using the sub-inguinal microscopic technique [13]. Only one (2.17%) short-term postoperative complication was reported, which was a superficial wound infection that was treated and adequately resolved with a cycle of oral antibiotics.

The median preoperative levels of FSH, LH, and total testosterone were in normal ranges (Table 2). Similarly, according to the WHO 2010 criteria [12], the median preoperative semen volume, motility, and total motile sperm count (TMSC) were within the reference values. However, the median preoperative semen concentration (median=12; IQR=7-21) was lower than expected, qualifying as oligospermia.

**Table 2:** Preoperative and postoperative hormone levels and semen parameters FSH: Follicle-Stimulating Hormone; LH: Luteinizing Hormone; TMSC: Total Motile Sperm Count

Variable	N	Preoperative, median (IQR)	N	Postoperative, median (IQR)	p-value
Hormones levels					
FSH (mlU/mL)	29	4.1 (2.6 – 6.7)	19	6.6 (3.1 – 8)	0.369
LH (mlU/mL)	23	4.6 (3.85 – 6.15)	14	3.9 (2.97 – 4.72)	0.077
Testosterone (ng/dL)	27	489 (372.5 – 651.5)	19	440 (372.5 – 537)	0.489
Semen parameters					
Volume (mL)	37	1.8 (1.1 – 3.5)	27	1.7 (1.5 – 3.05)	0.933
Concentration (mill/mL)	37	12 (7 – 21)	27	16 (10 – 26)	0.272
Motility (%)	37	46.5 (26.5 – 58.25)	27	48 (30 – 59)	0.826
TMSC	37	11.1 (2.28 – 23.4)	27	13.2 (5 – 30.5)	0.921

The median post-varicocelectomy hormone levels and semen parameters were also in the normal ranges (Table 2). An increase in the median sperm concentration was observed after surgery; however, the change was not statistically significant when compared to the preoperative value (p = 0.272). Overall, the median time between surgery and the first postoperative semen analysis was four (IQR= 3-6) months, and the median duration of follow-up was nine (IQR= 4-19) months. No statistically significant changes were observed when t-test analyses were performed comparing the pre- and post-varicocelectomy hormone levels and semen parameters (Table 2).

Of the 46 initial participants, 26 (56.52%) patients answered the questionnaire (Table 3). Despite our efforts, we were unable to establish contact with 18 patients, due to no response to phone calls. Additionally, two patients declined to participate in the study, without a specific reason. Table 1 shows that the interviewed patients were similar to the overall population, and no statistically significant differences were observed between both groups. The median age of the interviewed patients was 22 (IQR= 21-24) years, and the median time from surgery to the date of the interview was 36.5 (IQR= 18.25-62.25) months. The two patients who had initially consulted for infertility were able to naturally conceive after varicocelectomy. Of these 26 patients who answered the interview, seven reported having postoperative orchialgia, with five (71.41%) presenting these symptoms in the preoperative period. The other two patients (28.57%) developed testicular pain after surgery. Moreover, although three (11.53%) of the interviewed patients reported having been diagnosed with varicocele recurrence, none had undergone surgical reintervention. All patients denied having been diagnosed with hypogonadism or having used testosterone therapy.

**Table 3:** Responses to the questionnaire TRT: Testosterone replacement therapy

Question	Responses, n (%)
1. Orchialgia	
None	19 (73.08%)
Left	5 (19.23%)
Bilateral	2 (7.69%)
2. Paternity	
Yes	2 (7.69%)
No	0
Not interested	24 (92.31%)
3. Varicocele recurrence	
Yes	3 (11.53%)
No	23 (88.46%)
4. Surgical reintervention	
Yes	0
No	26 (100%)
5. Diagnosis of hypogonadism	
Yes	0
No	26 (100%)
6. Has used or is using TRT	
Yes	0
No	26 (100%)

## Discussion

Determining the optimal time to treat varicocele in adolescents is a subject for debate. Surgical options may theoretically cause future problems, and children and adolescents often lack the autonomy or maturity to make decisions that may impact their future fertility. The EAU Paediatric Guidelines in 2022 state that there is no evidence that surgical treatment of varicocele leads to improvement of andrological characteristics in the future if done in adolescence rather than adulthood [14]. However, Silay et al. demonstrated that pediatric varicocelectomy had a success rate defined by the disappearance of the varicocele, testicular recovery, and improvement in semen analysis from 87% to 100% [8]. Thus, the controversy primarily concerns the best age for surgery and whether varicocelectomy results suggest that patients should undergo surgery as adolescents or wait until adulthood. It is challenging to establish best practice guidelines in adolescents, given the scarce consensus on the surgical indications, short- and long-term outcomes, and patient satisfaction after varicocelectomy [5]. The aim of this study was to describe the adolescent population undergoing sub-inguinal microscopic varicocelectomy at a single institution and their changes in sexual hormone levels and semen parameters after surgery. Furthermore, we aimed to assess patient long-term satisfaction, paternity, and symptom improvement.

In the adult population, varicoceles are often treated in patients seeking to conceive children. For these patients, surgical indications are clearer, and paternity follow-up is more likely than for patients who are treated as adolescents [15]. We found that in adolescents, in contrast to adults, most patients (54.35%) sought medical treatment due to orchialgia. In the follow-up questionnaire, we observed that although testicular pain may improve after surgery, it was still reported in 71.41% of those interviewed with preoperative orchialgia. On the other hand, Peterson et al. evaluated 35 patients (mean age 25.7 years) after treatment, and 11% reported persistent orchialgia after varicocelectomy [16].

Another surgical indication that is exclusive to the pediatric and adolescent populations, but remains controversial, is testicular hypotrophy. In our study, 4.35% of the patients complained about this, and eight (17.39%) patients were found to have TVD at the physical exam. Li et al. conducted a meta-analysis that included 1,475 patients and concluded that patients with TVD > 10% benefited from varicocelectomy [17], as up to 70% would have testicular catch-up after surgery [18]. Nonetheless, TVD is still a controversial surgical indication as the different modalities to assess it (Prader or Rochester orchidometers, calipers, or ultrasound) are highly operator-dependent and lead to varying results [5].

Elevated testicular temperature and subsequent impairment of Leydig cell function with disturbance of testosterone biosynthesis are theoretical findings that have been discussed and well-characterized in varicocele pathogenesis [1,17]. However, in the pediatric population, the debate persists about whether sexual hormones would be affected and cause possible future repercussions. For instance, Fideleff et al. found that in adolescents, the presence of varicocele was not associated with differences in the levels of FSH, LH, or total testosterone [19]. Moreover, Zampieri et al. concluded the same for gonadotropins; however, they noticed elevated testosterone after treatment, which may have been due to the varicocelectomy itself or to the expected physiological development during puberty [20]. Our study also found normal gonadotropins and testosterone levels in patients pre- and post-varicocelectomy, without observing any statistically significant change after the procedure. Our questionnaire asked whether patients in the long-term after varicocelectomy had developed hypogonadism or were on TT, and responses were negative for both.

Multiple studies have examined the effects of varicocele on semen quality. A meta-analysis performed by Nork et al. demonstrated improvement in semen density and morphology after varicocelectomy [21]. Moreover, several studies performed in the pediatric population have also demonstrated an increase in sperm concentration after varicocelectomy [2,8,22,23]. For instance, an abnormal semen analysis represented the second most common complaint in our study (21.74%). We observed that the sperm median concentration before surgery was in the oligospermia range, and concentration improved to normal values after varicocelectomy. However, the difference was not statistically significant, possibly due to the sample size.

Varicocele is one of the main reasons that lead adult males to seek treatment for infertility [24]. Despite the difficulty of evaluating fertility in pediatric cases, Çayan et al. reported, through microsurgical repair, a paternity rate of 77.3% versus 48.4% and time to conception of 11.8 months versus 16.85 months, in treated and untreated adolescents, respectively [2]. In our study, two patients sought treatment due to infertility, and both were able to conceive after surgery. An important question is whether varicocele correction per se is a procedure generally capable of improving paternity rate or whether this benefit is only associated with the microsurgical technique used in our study, as Bogaert et al. evaluated the paternity rates after varicocele treatment with sclerotherapy and found no improvement [6].

We found only one short-term complication in our study, which was a superficial wound infection. In microscopic varicocelectomy, infection rates are very low (0.7%); consequently, systemic antibiotic prophylaxis is still debated [24]. Hydrocele is the most common complication in patients undergoing varicocelectomy, with rates of 0-12% [8]. However, none of the patients in our study developed or reported hydrocele, probably because the microsurgical technique has lower rates of hydrocele than inguinal or high inguinal (Palomo technique) and laparoscopic approaches [25]. Furthermore, three patients reported varicocele recurrence, but none of them reported surgical reintervention. As the questionnaire was done by phone, is difficult to determine whether the recurrence met clinical parameters. Lurvey et al. found the recurrence rate of varicocele after varicocelectomy to be 1.5-3.4% within a follow-up period of five years [26].

Our study is not without limitations. We evaluated the results after varicocelectomy specifically done with the sub-inguinal microscopic technique. Therefore, it is beyond the scope of this project to analyze symptoms in patients who underwent varicocelectomies that used other surgical approaches. In addition, we contacted patients by telephone, a survey method that led to a decrease in the number of respondents and made it impossible to perform physical examinations to assess testicular outcomes. Moreover, our sample size was small, and this could have precluded observing statistically significant differences. Long-term paternity was also difficult to evaluate as most of the interviewed patients were not actively trying to have children. Consequently, we believe that prospective, randomized controlled studies assessing semen parameters, hormone levels, and paternity rates between observational and interventional groups using different approaches and surgical techniques (e.g., artery sparing and non-artery sparing) might give a broader idea of the long-term outcomes and potential sequelae of this procedure in adolescents.

One of the main strengths of this study is that it has a long-term follow-up period, which allows for the evaluation of the long-term efficacy and safety of microscopic varicocelectomy in adolescents. Additionally, the study has well-defined inclusion criteria, which increases the homogeneity of the study population and reduces the potential for bias. Furthermore, the study uses validated outcome measures, such as semen analysis and sexual hormone levels, which increase the accuracy and reliability of the results. Overall, we provide valuable information on the long-term outcomes of microscopic varicocelectomy in adolescents and offer insight into its effectiveness and safety.

## Conclusions

Sub-inguinal microscopic varicocelectomy is a long-term, feasible, and safe procedure for well-selected adolescents, with spermatogenesis and hormone levels preserved after surgery. Patients should be counseled about the possibility of chronic pain and other complications such as hydrocele after surgery. Our study did not find a significant association between varicocelectomy and the development of hypogonadism. However, it is essential to interpret this finding with caution, as the sample size and study design may limit its generalizability to the broader population. Larger, prospective studies with a longer follow-up period are needed to generate results that will help build consensus in clinical practice and set appropriate expectations for adolescents after microscopic varicocelectomy.

## References

[1] Tanrikut C, Goldstein M, Rosoff JS, Lee RK, Nelson CJ, Mulhall JP (2011). Varicocele as a risk factor for androgen deficiency and effect of repair. BJU Int.

[2] Çayan S, Şahin S, Akbay E (2017). Paternity rates and time to conception in adolescents with varicocele undergoing microsurgical varicocele repair vs observation only: a single institution experience with 408 patients. J Urol.

[3] Kolon TF (2015). Evaluation and management of the adolescent varicocele. J Urol.

[4] Macey MR, Owen RC, Ross SS, Coward RM (2018). Best practice in the diagnosis and treatment of varicocele in children and adolescents. Ther Adv Urol.

[5] Elder J (2019). Does the evidence support adolescent varicocelectomy?. Eur Urol.

[6] Bogaert G, Orye C, De Win G (2013). Pubertal screening and treatment for varicocele do not improve chance of paternity as adult. J Urol.

[7] Verhovsky G, Neheman A, Rappaport YH, Kedem R, Hofman A, Zisman A, Haifler M (2018). Varicocele management strategies and resulting paternity rates in a cohort of young adults. Urology.

[8] Silay MS, Hoen L, Quadackaers J (2019). Treatment of varicocele in children and adolescents: a systematic review and meta-analysis from the European Association of Urology/European Society for Paediatric Urology guidelines panel. Eur Urol.

[9] Salzhauer EW, Sokol A, Glassberg KI (2004). Paternity after adolescent varicocele repair. Pediatrics.

[10] Pajovic B, Radojevic N (2013). Prospective follow up of fertility after adolescent laparoscopic varicocelectomy. Eur Rev Med Pharmacol Sci.

[11] Montague DK, Barada JH, Belker AM (1996). Clinical guidelines panel on erectile dysfunction: summary report on the treatment of organic erectile dysfunction. J Urol.

[12] (2022). World Health Organization. (‎2010)‎. WHO laboratory manual for the examination and processing of human semen, 5th ed. World Health Organization. WHO Laboratory Manual for the Examination and Processing of Human Semen, 5th Ed.

[13] Shiraishi K, Oka S, Matsuyama H (2016). Surgical comparison of subinguinal and high inguinal microsurgical varicocelectomy for adolescent varicocele. Int J Urol.

[14] (2023). Paediatric Urology: The guideline. Uroweb. Uroweb - European.

[15] Mason MM, Clavijo RI (2023). Does varicocele treatment in adolescence improve fertility outcomes in adulthood?. Eur Urol Focus.

[16] Peterson AC, Lance RS, Ruiz HE (1998). Outcomes of varicocele ligation done for pain. J Urol.

[17] Li F, Chiba K, Yamaguchi K (2012). Effect of varicocelectomy on testicular volume in children and adolescents: a meta-analysis. Urology.

[18] Moursy EE, ElDahshoury MZ, Hussein MM, Mourad MZ, Badawy AA (2013). Dilemma of adolescent varicocele: long-term outcome in patients managed surgically and in patients managed expectantly. J Pediatr Urol.

[19] Fideleff HL, Boquete HR, Suárez MG (2000). Controversies in the evolution of paediatric-adolescent varicocele: clinical, biochemical and histological studies. Eur J Endocrinol.

[20] Zampieri N, Ottolenghi A, Camoglio FS (2008). Painful varicocele in pediatric age: is there a correlation between pain, testicular damage and hormonal values to justify surgery?. Pediatr Surg Int.

[21] Nork JJ, Berger JH, Crain DS, Christman MS (2014). Youth varicocele and varicocele treatment: a meta-analysis of semen outcomes. Fertil Steril.

[22] Cayan S, Acar D, Ulger S, Akbay E (2005). Adolescent varicocele repair: long-term results and comparison of surgical techniques according to optical magnification use in 100 cases at a single university hospital. J Urol.

[23] Yamamoto M, Hibi H, Katsuno S, Miyake K (1995). Effects of varicocelectomy on testis volume and semen parameters in adolescents: a randomized prospective study. Nagoya J Med Sci.

[24] Richardson I, Nagler HM (2008). Systemic antibiotic prophylaxis not needed for microsurgical varicocelectomy. Urology.

[25] Al-Said S, Al-Naimi A, Al-Ansari A, Younis N, Shamsodini A, A-sadiq K, Shokeir AA (2008). Varicocelectomy for male infertility: a comparative study of open, laparoscopic and microsurgical approaches. J Urol.

[26] Lurvey R, Durbin-Johnson B, Kurzrock EA (2015). Adolescent varicocele: a large multicenter analysis of complications and recurrence in academic programs. J Pediatr Urol.

